# The Role of Societal Aspects in the Formation of Official COVID-19 Reports: A Data-Driven Analysis

**DOI:** 10.3390/ijerph18041505

**Published:** 2021-02-05

**Authors:** Marcell Tamás Kurbucz, Attila Imre Katona, Zoltán Lantos, Zsolt Tibor Kosztyán

**Affiliations:** 1 Department of Quantitative Methods, Faculty of Business and Economics, University of Pannonia, Egyetem Street 10, H-8200 Veszprém, Hungary; katona.attila@gtk.uni-pannon.hu (A.I.K.); kosztyan.zsolt@gtk.uni-pannon.hu (Z.T.K.); 2 Wigner Research Centre for Physics, Department of Computational Sciences, Konkoly-Thege Miklós Street 29-33, H-1121 Budapest, Hungary; 3 Research Centre of Faculty of Business and Economics, University of Pannonia, Egyetem Street 10, H-8200 Veszprém, Hungary; 4 Health Experience Institue, Közraktár Street 30-32, H-1093 Budapest, Hungary; zoltan.lantos@iask.hu; 5 Institute of Advanced Studies (iASK), Chernel Street 14., H-9730 Kőszeg, Hungary; 6 MTA-PE Budapest Ranking Research Group, Egyetem Street 10., H-8200 Veszprém, Hungary

**Keywords:** COVID-19, country reports, socioeconomic analysis, data-driven approach

## Abstract

This paper investigates the role of socioeconomic considerations in the formation of official COVID-19 reports. To this end, we employ a dataset that contains 1159 pre-processed indicators from the World Bank Group *GovData360* and *TCdata360* platforms and an additional 8 COVID-19 variables generated based on reports from 138 countries. During the analysis, a rank-correlation-based complex method is used to identify the time- and space-varying relations between pandemic variables and the main topics of World Bank Group platforms. The results not only draw attention to the importance of factors such as air traffic, tourism, and corruption in report formation but also support further discipline-specific research by mapping and monitoring a wide range of such relationships. To this end, a source code written in R language is attached that allows for the customization of the analysis and provides up-to-date results.

## 1. Introduction

Research on the COVID-19 pandemic has grown rapidly since the outbreak of the disease; however, despite the enormous media attention on countries’ reports, only a few articles address the number of officially reported cases and deaths as a social phenomenon. As many studies have pointed out, there is a significant discrepancy between the officially confirmed data and recently published estimates (see, e.g., [1,2]). However, what do these data reflect, beyond the true nature of the virus? Of the few articles dealing with this question, authors [3] examined the protective effect of BCG (Bacillus Calmette–Guérin) vaccine on COVID-19 infections and the death toll while using indicators such as the Human Development Index (HDI), per-capita GDP, and urban population percentage as additional control variables. Moreover, in line with [4], they applied the Corruption Perception Index (CPI) as a proxy for the reliability of reported COVID-19 data. Other authors [5] found that measures of globalization, related to the migrant stock and air travel, were positively associated with both total cases and deaths. Results [6] suggest that more equitable access to healthcare does indeed increase testing rates and lower the death rate. Authors in [7] showed that more democratic political institutions experienced deaths on a larger per-capita scale and sooner than did less democratic countries, and based on [8], the population size and government health expenditure are strongly related to COVID-19 cases.

In contrast to these (mostly) discipline-specific studies, our goal is to map, analyze, and monitor a wide range of such relationships in time and space by applying a data-driven approach. To provide a deeper understanding of the role of societal aspects in the formulation of COVID-19 reports, we employ a rank-correlation-based complex method and a dataset that contains 1159 pre-processed indicators from the World Bank Group *GovData360* and *TCdata360* platforms and an additional 8 COVID-19 variables. The results not only draw attention to the importance of factors such as air traffic, tourism, and corruption in report formation but also support decision makers and discipline-specific research by providing a source code written in R language (in R Notebook) that allows for the customization of the analysis and provides up-to-date results.

The paper is organized as follows. Section 2 introduces the data and methodology used during the calculations. Section 3 presents and discusses the results. Section 4 contains the measurement error analysis of the results. Finally, Section 5 provides the conclusions and proposes future research directions.

## 2. Data and Methodology

### 2.1. Joint Dataset of GovData360, TCdata360, and COVID-19 Reports

This paper follows the steps of [9] when creating a linked database of governance, trade, and competitiveness indicators with COVID-19 reports. (To derive the up-to-date dataset, we use the author’s R source code, which is publicly available at [10]). Former indicators were obtained from the *GovData360* and *TCdata360* platforms using the *data360r* (version: 1.0.8) R package [11]. From these platforms, only annual indicators from 2015 and later were collected, and their missing values were replaced with previous annual values in descending order by year until 2015. During pre-processing, indicators (columns) where the ratio of missing values exceeded 50% were filtered out. Then, the same filtration was applied above 25% in the case of countries (rows). Finally, highly correlated variables and variables with near-zero variances were removed, and the standardized form of the retained 1159 indicators was connected with 8 COVID-19 variables, generated on the basis of the official reports of 138 countries [12]. Note that auxiliary indicators measuring the number of data sources and standard error were also filtered out, and variables with near-zero variances were eliminated using the default settings of *nearZeroVar* function contained by the *caret* (version: 6.0-85) R package [13]. The presented data were compiled on 22 July 2020. Table 1 shows the description of the variables in the structure of the final dataset.

### 2.2. Community-Based Model Reduction

Our goal is to map the time- and space-varying relationship between COVID-19 (furthermore dependent) variables ($Y:=\{y_{1},..,y_{M}\}$) and indicators from *GovData360* and *TCdata360* platforms (furthermore independent variables, $X:=\{x_{1},..,x_{N}\}$). To obtain an easily interpretable, comprehensive picture from these connections, similar *GovData360* and *TCdata360* indicators are grouped and characterized by latent variables. The applied steps are as follows.

First, standardized independent variables that have higher absolute (Spearman) rank correlation than an α parameter with at least one dependent variable are selected and denoted as X⊆X. Formally:

(1) X:={x|x∈X∧∃y∈Y,where|cor(y,x)|≥α}.

Then, the rank correlation matrix of the selected variables is used as an adjacency matrix A, in which absolute rank correlation values below a β parameter are substituted by 0. Formally:

(2) $$a_{ij}={\left[A\right]}_{ij}:=\left\{\begin{array}{cc} 0, & \;\mathrm{if}\;\;\;|cor(x_{i},x_{j})|<\beta \\ |cor(x_{i},x_{j})|, & \;\mathrm{if}\;\;\;|cor(x_{i},x_{j})|\geq \beta \end{array}\right.,\;x_{i},x_{j}\in X.$$

Note that the adjacency matrix A defines a network, where the vertices are the selected variables (V=X), edges are indicated by the nonzero values ($e_{ij}\in E\Leftrightarrow {\left[A\right]}_{ij}=a_{ij}>0$), and their weight is the absolute rank correlation between the selected variables ($w:E\to R^{+},w\left(i,j\right)=a_{ij}$). (Note that the same strategy was applied by, e.g., [15,16], to visualize variable similarity.)

To group similar variables, our goal is to separate this network into groups of vertices that have fewer connections between them than inside the communities. In the literature, this task [17] is referred to as modularity-based community analysis (see, e.g., [18]) or simply community detection (see, e.g., [19]). Although the proposed method may seem more complicated than traditional model reduction methods, they cannot be used because of the large number of variables and relatively few observations. In addition, the visualization of the (correlation) network facilitates control over community formation (especially if *N* is large). This benefit is realized by using the Louvain community detection method [20] with an associated filtrating procedure that gains heterogeneity between the groups by eliminating weakly connected group members.

After Louvain community detection, we obtain $C:=\{c_{1},..,c_{n}\}$ communities on G(V,E), which specifies {$G^{c_{1}},..,G^{c_{n}}\}=G$ partitions of network *G*. As a next step, each community is represented by a single composite (so-called latent) variable (${\hat{x}}^{c_{i}}$) obtained by the weighted linear combination of member variables:

(3) $${\hat{x}}^{c_{i}}=\frac{\sum_{j}x_{j}e_{j}}{\sum_{j}e_{j}},j\in V^{c_{i}},i=1,2,..,n,$$

where

(4) $$e_{j}=\frac{1}{\lambda }\sum_{t\in V^{c_{i}}}a_{jt}e_{t}$$

is the eigenvector centrality of node *j*, and λ>0 is a constant. Louvain modularity and eigenvector centrality were calculated using the *cluster_louvain* and *eigen_centrality* functions of *igraph* (version: 1.2.4.2) R package, respectively [21]. Note that the use of eigenvector centrality as a weight ensures that deeper embedded variables (within the given community) play a greater role in the formation of the latent variable. (Also note that the use of standardized independent variables results in standardized latent variables). To increase the homogeneity of communities, we calculate the absolute (Spearman) rank correlation of each variable within the community *i* with the related latent variable ${\hat{x}}^{c_{i}}$, and variables that have weaker absolute rank correlation than a γ parameter are removed from their communities. Finally, the steps of this paragraph (from community detection to filtration) are repeated until no more variables can be eliminated. Note that while the proposed algorithm finds strongly interrelated indicators, correlations between modules still can exist. Thus, completely independent communities are not guaranteed. Although the use of factor analysis with orthogonal rotation may result in independent communities, as there are more observations than variables in the studied dataset, the use of this method is not recommended.

**Figure 1:**
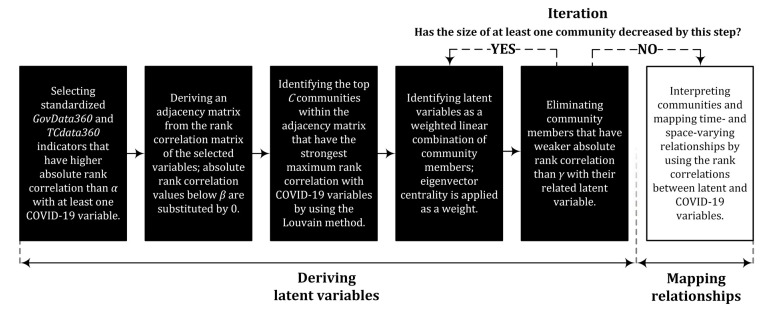
Calculation steps. The calculation process can be divided into six steps. The first five steps are responsible for creating communities characterized by latent variables, and the last one is about the interpretation of the communities and mapping their relationships with COVID-19 variables.

At the end of the process, we rank communities (characterized by latent variables) by their absolute rank correlation with dependent variables. Then, we select the top C≤n interpretable communities and investigate their relationship with the dependent variables through their absolute rank correlation coefficients. To examine the regional differences in addition to the study of time-varying relationships, these correlations are identified as well on the subset of different regions (see variable $r^{*}$ in Table 1). The calculation steps are summarized in Figure 1.

## 3. Results and Discussion

Following the calculation order presented above, we first illustrate and interpret the results of community detection to identify the most important topics reflected in official COVID-19 reports. Then, we investigate the time- and space-varying relations of these communities with different pandemic variables.

### 3.1. Topics Most Related to COVID-19 Reports

**Figure 2:**
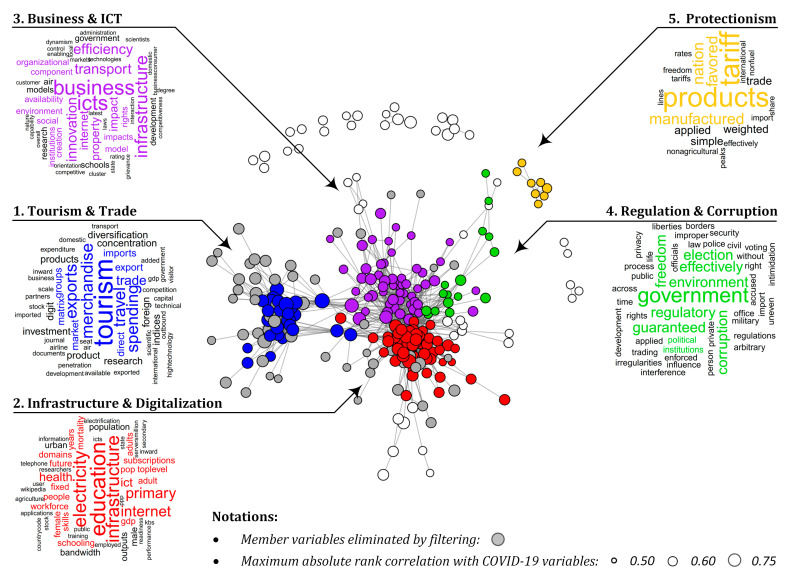
Detected communities. The five communities of correlated *GovData360* and *TCdata360* indicators. Indicators are denoted by vertices and the edges representing the strong correlations among them. The interpretation of communities is supported by word clouds generated on the basis of the names of member indicators. Note that the applied method may not create purely separable communities, so the same words can occur in different word clouds. This can also occur if the same word appears in the names of significantly different indicators.

When setting α, β, and γ parameters, our goal was to group the widest possible range of important *GovData360* and *TCdata360* indicators without obtaining communities that are difficult to interpret. To accomplish this goal, we set the parameters as α=0.535, β=0.828, and γ=0.770, which resulted in a network containing 319 indicators (vertices) and 1669 edges, representing the strong correlations among the indicators. Figure 2 illustrates five communities (C=5) detected within this network and helps their interpretation with word clouds generated on the basis of the names of member indicators. Word clouds were constructed after text cleaning and pre-processing by using the *wordcloud* (version: 2.6) R package [22]. Since pre-processing was based on a frequency list of terms contained by the names of the indicators, overlapping terms may occur in the composed word clouds. For example, the term “infrastructure” is included in several indicator names such as *Electricity and telephony infrastructure* and *Quality of air transport infrastructure*, even though they relate to different aspects of infrastructure.

As Figure 2 shows, the topics most related to official COVID-19 reports are (1) *tourism and trade*, (2) *infrastructure and digitalization*, (3) *business and ICT*, (4) *regulation and corruption*, and (5) *protectionism*. From these, *tourism and trade* associates with the flow of people and goods, as reflected by the most frequent terms such as *tourism*, *travel*, *merchandise*, and *imports*. The contribution of the database also confirms this finding since most indicators of this community are part of the *World Travel & Tourism Council* (27%), *United Nations Conference on Trade and Development Statistics* (19%), and *World Integrated Trade Solution* (15%) datasets. The second community describes the infrastructure, especially in the field of digitalization, including variables such as *ICT access*, *public services*, and *secure internet servers/million pop*. Most of these variables are derived from the *World Economic Forum Global Competitiveness Index* (34%), *Global Innovation Index* (24%), and *World Development Indicators* (17%) databases. The third community, so-called *business and ICT*, is adjacent to *infrastructure and digitalization*. As its name suggests, it is in connection with information and communication technology (ICT); however, this community focuses more on business aspects such as *innovation*, *efficiency*, and *competitiveness*. The group *regulation and corruption* includes variables such as *regulatory quality*, *political environment*, and *corruption*. The sources of most of these variables are the *World Justice Project—Rule of Law* (31%), Global Innovation Index (25%), and Global State of Democracy (19%). Finally, the fifth community is labeled *protectionism* because all of its variables are related to *tariffs*. The variables for each community are detailed in the Supplementary Materials.

### 3.2. Relations with COVID-19 Reports

**Figure 3:**
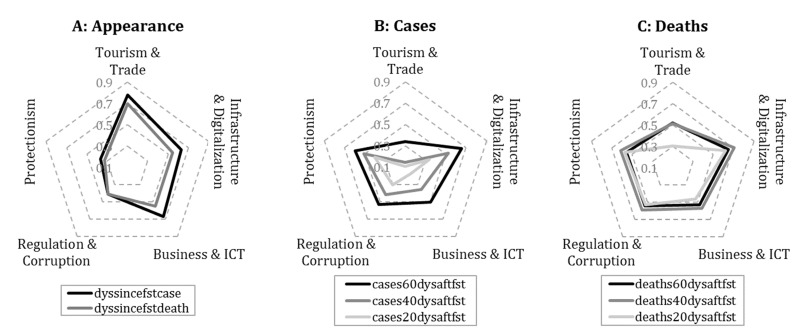
Absolute rank correlation between COVID-19 and latent variables. The correlations are presented according to the different COVID-19 variables related to the appearance of the virus (**A**), the number of cases (**B**), and the death toll (**C**).

To visualize the absolute rank correlation between the COVID-19 variables and the communities characterized by latent variables, radar charts are employed. In Figure 3, these relations are classified into three groups. The first focuses on the time elapsed since the first registered data, while the other two relate to the officially reported cases and deaths per capita aggregated by using different time windows.

As Figure 3 shows, indicators measuring the appearance of the virus are strongly correlated with tourism- and trade-related activities. Taking a closer look at the standalone variables within this community, *GCI 4.0: Air transport* and *outbound travel and tourism expenditure* have the strongest (Spearman) rank correlation coefficients with the days passed since the first case was reported (0.789 and 0.777, respectively). Moreover, this COVID-19 indicator has a strong connection with variables of international trade as well, such as *Merchandise: Trade matrix by product groups, imports* (0.755), *Index Of Export Market Penetration* (0.736), and *Number of export partners* (0.707) from the same community. In light of these close relationships, it may be surprising to find that this community has a relatively weak connection with the reported number of cases and deaths; however, increased controls at airports and the rapid closure of borders could be reflected in this result.

In contrast to *tourism and trade*, these COVID-19 indicators are closely linked to the other four communities, especially to *infrastructure and digitalization*. From this community, variables related to digital development such as *fixed broadband subscriptions (per 100 people)* and *online creativity* show the strongest positive (Spearman) rank correlation with the number of deaths per capita (0.659 and 0.653, using the 60-day time window), which suggests that a significantly higher death toll has been reported by more developed countries. It is also reflected by the positive correlation of this COVID-19 variable with *A. Health* indicator calculated from healthy life expectancy (0.582) as well as by its strong negative relationship with *GCI 4.0: Exposure to unsafe drinking water* (−0.676).

On the basis of these results, while the data suggesting the appearance of the virus seem to be reliable and relatively easy to explain, reports on cases and deaths appear highly distorted. On the one hand, this distortion may be a consequence of the poor health infrastructure that makes measurement difficult, but on the other hand, political interests could also be tied to underreporting. Since the *regulation and corruption* community’s *regulatory quality* and *freedom from corruption score* indicators have a strong positive correlation with the reported number of cases (0.560 and 0.533, respectively) and deaths (0.565 and 0.523, respectively, using the 60-day time window), the reports of countries with higher levels of corruption seem much less authentic. Furthermore, detected communities contain strikingly many indicators related to the development of the information society, which counteracts disinformation.

To support discipline-specific research, we detailed the correlations of each member variable with different COVID-19 indicators in the Appendix A. These correlations can provide a deeper understanding of phenomenons mapped by using latent variables. For instance, the strongest correlations were found between the spread of the virus and the latent variable of the *tourism and trade* community. On the basis of Table A1, the strength of these relationships mainly is due to the indicators such as *GCI 4.0: Air transport*, *International tourism and number of arrivals*, and *Outbound Travel & Tourism Expenditure*, or more generally, due to the number of inbound and outbound travels. Similar to latent variables (see Figure 3), these standalone indicators typically show increasing correlations with time window expansion; however, this change over time can vary significantly from region to region.

### 3.3. Regional Differences

**Figure 4:**
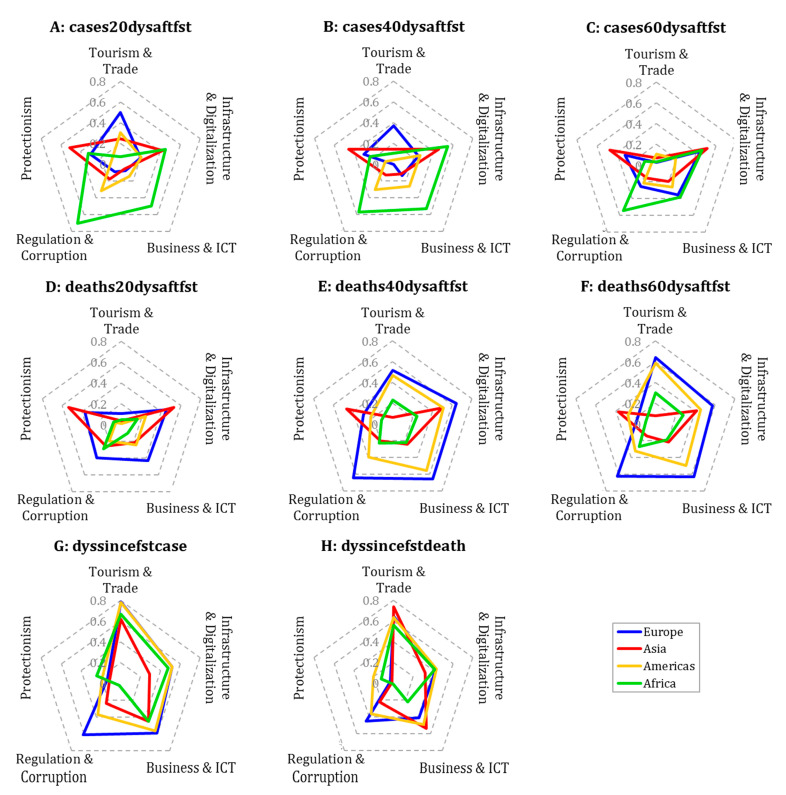
Absolute rank correlation between COVID-19 and latent variables by region. The correlations are presented according to the different COVID-19 variables that measure the total number of cases/deaths per capita after 20 days (**A**/**D**), 40 days (**B**/**E**), and 60 days (**C**/**F**) from the first case/death, as well as the number of days since the first case/death (**G**/**H**).

To examine how the results presented in the previous subsection differ from region to region, countries are divided into four groups by using the *region ID* variable (denoted as $r^{*}$ in Table 1). These groups are *Europe*, *Asia*, *Americas*, *Africa*, and *Oceania*; however, the last group was omitted from the investigation due to its small sample size (two countries). The regional differences in the relations of COVID-19 and latent variables are presented in Figure 4.

On the basis of Figure 4, we can conclude that the impact of *tourism and trade* on the spread of the virus is significant regardless of region; however, the variable measuring the appearance of the first case shows the highest (Spearman) rank correlation with this community in the *Americas* and *Europe*. In these two regions, variables such as *GCI 4.0: Air transport* (0.803 and 0.791, respectively) and *government spending on travel and tourism service* (0.881 and 0.715, respectively) have one of the highest correlations with days elapsed since the first case. Moreover, in the *Americas* and *Europe*, this community, and especially its tourism- and air-transport-related indicators, shows an increasingly close relationship with the number of registered deaths per capita as the time window expands. Accordingly, regulations on foreign travel restrictions and airport controls are particularly important in these regions.

Next to the *Americas* and *Europe*, in *Asia*, variables measuring the spread of the virus are also strongly tied to the *tourism and trade* community, but these variables have a stronger rank correlation with the data related to first death. Unlike other regions, reports from *Asian* countries are mostly related to *infrastructure and digitalization* and *protectionism* communities; however, even these relations appear weak in comparison with the relationships detected in other regions. To obtain stronger ties, it may be worthwhile to map the topics that contain the most important variables separately for this region.

Finally, reported data both in *Africa* and *Europe* have remarkably close connections with the *regulation and corruption* community, especially with indicators such as *political environment* and *freedom from corruption score*. While in *Africa* these variables are typically related to reported case numbers (0.568 and 0.551), in *Europe* they show a stronger correlation with deaths (0.495 and 0.583, using the 60-day time window, respectively), which suggests that the reports of these regions are less credible. Note that the correlations of standalone variables calculated on different regional subsamples are contained in the Supplementary Materials.

## 4. Measurement Error Analysis

As it was discussed in Section 3.2, while the data suggesting the appearance of the virus seem to be reliable and relatively easy to explain, reports on cases and deaths appear highly distorted. Since these measurement errors can affect the community detection outcomes through the distorted rank correlation coefficients, in this section, we conduct a simulation to analyze the validity of our results under the presence of measurement errors.

During the simulation, by using the multiplicative measurement error model, we added random measurement errors to the dependent variables and conducted all the calculation steps described by Figure 1. The applied measurement error model is as follows:

(5) $$y_{i}^{*}=y_{i}\varepsilon_{i},$$

where the reported and the masked dependent variables are denoted by $y_{i}$ and $y_{i}^{*}$, and $\varepsilon_{i}$ is an independent random variable following a normal distribution with a mean 1 and standard deviation $\sigma_{\varepsilon }$. During the simulation, $y_{i}^{*}$ was estimated by using different $\sigma_{\varepsilon }$ values, then the resulted communities were examined. In order to characterize the structure of the communities we investigate the number of vertices and edges to the correlation network of the variables, number of communities found, and number of included variables in each community (see Table 2).

Although number of extracted communities does not change significantly when $\sigma_{\varepsilon }\geq 0.1$, both the structure of the correlation network and the sizes of the communities start to vary. On the basis of this additional calculation, communities presented in Section 3.1 are stable in the event of a small or moderate measurement error.

## 5. Conclusions and Future Work

Although some of the recent studies have already investigated the relationship of COVID-19 data with different socioeconomic indicators, the role of societal considerations in the formation of official COVID-19 data is not yet clear. In contrast to these studies, our goal was to map, analyze, and monitor a wide range of such relationships in time and space by applying a data-driven approach. To this end, we employed a rank-correlation-based complex method and a dataset that contains 1159 pre-processed indicators from the World Bank Group *GovData360* and *TCdata360* platforms and an additional 8 COVID-19 variables generated on the basis of the officially reported number of cases and deaths.

From our results, the topics most related to official COVID-19 reports are *tourism and trade*, *infrastructure and digitalization*, *business and ICT*, *regulation and corruption*, and *protectionism*. By examining these topics and the variables they compress, we found that tourism- and air-transport-related variables are key factors in the spread of the virus, especially in the *Americas* and *Europe*. In these two regions, the variables of the *tourism and trade* community show close connections with the reported death toll as well, which also emphasizes the importance of regulations on foreign travel restrictions and airport controls. In addition, the number of reported cases and deaths seems unreliable since developed countries generally reported more cases and deaths than developing countries. In line with the results, the two possible reasons for underreporting may be the poor health infrastructure that makes measurement difficult and the political will that is opposed to exploring and presenting the real epidemiological situation. Accordingly, we experienced the closest relationship between the level of corruption and reported data in *Europe* and *Africa*.

Using the proposed analysis, further interesting regional and temporal patterns can be identified, as the data will be updated over time. To support this research, we attach an R Notebook file (see Supplementary Materials) that not only updates the dataset but is also able to conduct all the analysis steps, including variable filtering and the compilation of figures. As a further advantage, this source code can be easily customized and allows researchers to apply arbitrary time frames during the analysis. Finally, in the Appendix A, we provide all the relationships identified during the analysis to support discipline-specific investigations.

## Figures and Tables

**Table 1:** Variable description.

Note	Group		Description and Short Name of Variables	NA	Source
$c^{*}$	ID		Country ID; $c^{*}\in \left\{1,2,\ldots ,138\right\}.$	0%	a
$r^{*}$			Region ID; $r^{*}\in \left\{1,2,\ldots ,5\right\}.$	0%	b
$y_{1}$	COVID-19	Cases	The number of days since the first case. (**dyssincefstcase**)	0%	a
$y_{2}$			The total number of cases per capita after 20 days from the first case. (**cases20dysaftfst**)	0%	a
$y_{3}$			The total number of cases per capita after 40 days from the first case. (**cases40dysaftfst**)	0%	a
$y_{4}$			The total number of cases per capita after 60 days from the first case. (**cases60dysaftfst**)	0%	a
$y_{5}$		Deaths	The number of days since the first death. (**dyssincefstdeath**)	0%	a
$y_{6}$			The total number of deaths per capita after 20 days from the first death. (**deaths20dysaftfst**)	1%	a
$y_{7}$			The total number of deaths per capita after 40 days from the first death. (**deaths40dysaftfst**)	1%	a
$y_{8}$			The total number of deaths per capita after 60 days from the first death. (**deaths60dysaftfst**)	3%	a
$x_{k}$		Gov	The complete list of standardized *GovData360* and *TCdata360* indicators is contained in the Supplementary Materials; k∈{1,2,…,500}, l∈{501,502,…,1159}.	5% *	c
$x_{l}$		TC		6% *	c

* Average missing value ratio. a: [12], the population data are from [14]. b: Own categories based on the country data of [12]. c: [11].

**Table 2:** Results of the measurement error analysis.

$\sigma_{\varepsilon }$	Number of...		C	Size of Community...				
	Vertices	Edges		1	2	3	4	5
0.00 *	321.0	1656.0	5.0	26.0	58.0	55.0	13.0	7.0
0.01	313.7	1655.9	5.0	26.0	57.7	55.8	13.2	7.0
0.03	309.5	1638.2	5.0	26.0	56.1	56.3	13.2	7.0
0.05	298.5	1594.7	5.0	26.0	55.7	51.9	13.2	7.0
0.10	275.8	1515.4	4.9	45.1	35.5	43.3	13.1	7.0
0.15	202.7	1173.0	5.0	47.3	17.0	23.3	15.0	7.0
0.20	208.3	1203.1	4.6	49.6	19.8	25.1	10.9	7.0
0.30	186.0	1049.0	5.2	36.0	17.7	18.4	16.4	9.4

The calculation parameters used are as follows: α = 0.535, β = 0.828 and γ = 0.770. * Original case, where *y*_i_ = $y_{i}^{*}$.

## Data Availability

Data and source code are available in the Supplementary Material.

## Supplementary Materials

The following are available online at https://www.mdpi.com/1660-4601/18/4/1505/s1. File B.1: Metadata. The metadata of *GovData360* and *TCdata360* indicators used. File B.2: Regional correlations. Standalone correlations in the regional dataset. File B.3: Source of COVID-19 data. The source of the COVID-19 dataset collected by [13]. According to the place of their publication, these sources are websites of ministries of health (43%), websites of public health institutes (9%), websites from other national authorities (6%), WHO websites, and WHO situation reports (2%), and official dashboards (10%). Besides, ECDPC screens social media accounts maintained by national authorities, for example Twitter, Facebook, YouTube, or Telegram accounts run by ministries of health (28%) and other official sources (e.g., official media outlets) (2%). File B.4: Data generation and analysis. Datasets were generated and analyzed with R Notebook, which can be used to update datasets and customize the analyses.

## Appendix A. Standalone Correlations

**Table A1:** Standalone correlations in the *tourism and trade* community. Spearman rank correlations between COVID-19 variables and indicators of the *tourism and trade* community in the worldwide dataset.

Name	Type	dyssincefstcase	cases20dysaftfst	cases40dysaftfst	cases60dysaftfst	dyssincefstdeath	deaths20dysaftfst	deaths40dysaftfst	deaths60dysaftfst
Trade, competition, & market scale	rank *	0.69	−0.15	0.07	0.28	0.68	0.19	0.42	0.43
Scientific and technical journal articles	value	0.77	−0.19	0.07	0.25	0.69	0.20	0.43	0.44
Travel and Tourism direct contribution to GDP	usd nominal	0.75	−0.28	−0.04	0.15	0.66	0.08	0.32	0.35
Outbound Travel & Tourism Expenditure	usd nominal	0.78	−0.19	0.04	0.25	0.70	0.18	0.41	0.44
Index Of Export Market Penetration	value	0.74	−0.19	0.06	0.27	0.71	0.30	0.51	0.53
Merchandise: Trade matrix by product groups, imports	value	0.76	−0.27	−0.01	0.20	0.67	0.16	0.40	0.41
Government spending on travel and Tourism service	usd nominal	0.67	−0.18	0.05	0.23	0.68	0.17	0.41	0.42
Capital investment in Travel and Tourism	usd real	0.71	−0.21	−0.03	0.15	0.62	0.09	0.32	0.35
Services, etc., value added (current US$)	value	0.70	−0.27	−0.02	0.17	0.66	0.08	0.33	0.36
Business Tourism Spending	usd_nominal	0.74	−0.29	−0.06	0.15	0.64	0.08	0.32	0.37
Number of export partners	value	0.71	−0.15	0.08	0.29	0.66	0.32	0.51	0.53
Foreign Direct Investment: Inward stock	percentage of world	0.69	−0.14	0.09	0.28	0.65	0.20	0.42	0.45
Domestic Tourism Spending	usd nominal	0.70	−0.36	−0.12	0.08	0.63	0.04	0.29	0.34
Available airline seat	rank *	0.76	−0.22	0.00	0.20	0.64	0.11	0.33	0.35
High-technology exports (current US$)	value	0.69	−0.14	0.08	0.27	0.63	0.25	0.45	0.45
Visitor Exports (Foreign spending)	usd real	0.74	−0.11	0.10	0.27	0.66	0.23	0.42	0.43
No. Of Imported HS6 Digit Products	value	0.68	−0.17	0.06	0.27	0.66	0.29	0.50	0.51
Citable documents H index	rank *	0.71	−0.19	0.06	0.25	0.67	0.25	0.47	0.49
No. Of Exported HS6 Digit Products	value	0.65	−0.13	0.10	0.30	0.64	0.32	0.51	0.52
GCI 4.0: Air transport	score	0.79	−0.07	0.16	0.35	0.66	0.28	0.47	0.46
International tourism, number of arrivals	value	0.72	−0.16	0.05	0.24	0.64	0.18	0.40	0.43
Research and development (R&D)	rank *	0.72	0.01	0.23	0.39	0.65	0.36	0.53	0.51
GCI 4.0: Research	rank *	0.72	0.03	0.26	0.42	0.62	0.37	0.55	0.54
Merchandise: Concentration and diversification indices of exports by country	diversification index *	0.63	−0.04	0.18	0.34	0.60	0.39	0.56	0.53
Merchandise: Trade matrix by product groups, exports	value	0.62	−0.28	−0.06	0.12	0.56	0.09	0.32	0.34
Merchandise: Concentration and diversification indices of imports by country	diversification index *	0.67	0.00	0.23	0.42	0.64	0.38	0.57	0.58

* The correlations of the rank variables and diversification indices were multiplied by −1.

**Table A2:** Standalone correlations in the *infrastructure and digitalization* community. Spearman rank correlations between COVID-19 variables and indicators of the *infrastructure and digitalization* community in the worldwide dataset.

Name	Type	dyssincefstcase	cases20dysaftfst	cases40dysaftfst	cases60dysaftfst	dyssincefstdeath	deaths20dysaftfst	deaths40dysaftfst	deaths60dysaftfst
Public Services	score *	0.59	0.38	0.53	0.65	0.49	0.60	0.67	0.61
ICT access	rank *	0.59	0.38	0.54	0.65	0.51	0.63	0.69	0.62
Country rank and value in the UNCTAD B2C E-commerce Index	rank *	0.61	0.30	0.46	0.59	0.52	0.59	0.67	0.61
3rd pillar: Infrastructure	rank *	0.56	0.33	0.49	0.62	0.50	0.60	0.69	0.64
Secure Internet servers/million pop.	rank *	0.53	0.38	0.52	0.64	0.48	0.63	0.69	0.65
Fixed broadband subscriptions (per 100 people)	value	0.56	0.30	0.50	0.61	0.53	0.61	0.71	0.66
GCI 4.0: Exposure to unsafe drinking water	score *	−0.57	−0.34	−0.53	−0.66	−0.53	−0.64	−0.72	−0.68
GCI 4.0: 6.B Future workforce	score	0.59	0.33	0.51	0.64	0.52	0.59	0.67	0.60
B. Readiness subindex	value	0.61	0.30	0.49	0.62	0.51	0.60	0.69	0.63
B. Electricity and telephony infrastructure	rank *	0.56	0.35	0.50	0.62	0.50	0.58	0.66	0.61
5th pillar Higher education and training	value	0.61	0.28	0.45	0.57	0.60	0.54	0.64	0.57
GDP per person employed (constant 2011 PPP $)	value	0.60	0.33	0.50	0.64	0.55	0.59	0.69	0.65
Infrastructure	rank *	0.65	0.24	0.44	0.59	0.57	0.52	0.64	0.60
Information and communication technologies (ICTs)	score (0–100)	0.67	0.19	0.40	0.56	0.60	0.50	0.62	0.58
GCI 4.0: International co-inventions	score	0.61	0.27	0.44	0.56	0.54	0.55	0.64	0.59
GCI 4.0: Pillar 2: Infrastructure	rank *	0.71	0.20	0.43	0.59	0.61	0.54	0.66	0.62
5th pillar: Skills	value	0.58	0.32	0.47	0.59	0.51	0.57	0.63	0.56
GNI per capita (constant 2010 US$)	value	0.57	0.33	0.48	0.64	0.54	0.60	0.70	0.66
4th pillar Health and primary education	value	0.60	0.29	0.45	0.57	0.52	0.56	0.61	0.53
GCI 4.0: Internet users	rank *	0.62	0.33	0.50	0.63	0.50	0.60	0.66	0.61
Wikipedia monthly edits	value	0.50	0.34	0.54	0.67	0.51	0.69	0.75	0.71
GCI 4.0: Pillar 3: ICT adoption	rank *	0.61	0.30	0.44	0.56	0.44	0.53	0.59	0.53
ICT PCT patents, applications/million pop.	rank *	0.62	0.18	0.38	0.51	0.53	0.50	0.61	0.56
GCI 4.0: Electricity infrastructure	rank *	0.65	0.28	0.46	0.56	0.52	0.52	0.57	0.50
Online creativity	score (0–100)	0.51	0.33	0.50	0.60	0.46	0.62	0.70	0.65
Researchers	rank *	0.63	0.14	0.37	0.52	0.53	0.57	0.67	0.61
Mortality rate, adult, female (per 1000 female adults)	value	−0.65	−0.35	−0.53	−0.65	−0.55	−0.60	−0.66	−0.61
Human capital and research	score (0–100)	0.62	0.18	0.35	0.50	0.54	0.49	0.61	0.56
Quality of electricity supply	1–7 best	0.57	0.28	0.42	0.55	0.50	0.53	0.62	0.58
Legitimacy of the State	score *	0.61	0.21	0.40	0.54	0.50	0.50	0.61	0.58
GCI 4.0: Mean years of schooling	rank *	0.50	0.34	0.48	0.57	0.44	0.54	0.61	0.55
GCI 4.0: Trademark applications	score	0.53	0.36	0.53	0.63	0.50	0.60	0.68	0.64
Generic top-level domains (gTLDs)	score (0–100)	0.49	0.33	0.48	0.59	0.51	0.58	0.68	0.63
Foreign Direct Investment: Inward stock	usd per capita	0.42	0.43	0.54	0.63	0.37	0.60	0.63	0.58
GCI 4.0: Skills of future workforce	rank *	0.57	0.32	0.48	0.60	0.48	0.55	0.60	0.52
Int’l Internet bandwidth, kb/s per user	value	0.48	0.38	0.53	0.65	0.47	0.62	0.70	0.67
GCI 4.0: Electrification rate	rank *	0.57	0.35	0.53	0.61	0.50	0.60	0.66	0.59
Secondary education gross enrollment rate, %	rank *	0.52	0.31	0.52	0.65	0.52	0.59	0.67	0.63
Mean years of schooling	scale (0 to 1)	0.49	0.29	0.45	0.57	0.47	0.54	0.62	0.56
Fixed telephone subscriptions (per 100 people)	value	0.48	0.36	0.52	0.62	0.51	0.60	0.67	0.63
Internet bandwidth	rank *	0.46	0.43	0.54	0.62	0.40	0.62	0.66	0.61
Self-employed, total (% of total employment)	percent	−0.50	−0.35	−0.46	−0.60	−0.40	−0.56	−0.63	−0.60
School life expectancy	rank *	0.50	0.25	0.45	0.58	0.50	0.58	0.67	0.63
Creative outputs	rank *	0.59	0.24	0.42	0.53	0.51	0.49	0.60	0.54
Environmental performance	index	0.53	0.29	0.48	0.60	0.54	0.57	0.66	0.61
Agriculture, value added (% of GDP)	value	−0.46	−0.34	−0.46	−0.60	−0.42	−0.54	−0.61	−0.58
A. Health	rank *	0.59	0.30	0.48	0.60	0.57	0.55	0.62	0.58
Innovation Output Sub-Index	rank *	0.63	0.13	0.33	0.46	0.54	0.46	0.59	0.56
GCI 4.0: Pupil-to-teacher ratio in primary education	score	0.51	0.43	0.59	0.67	0.43	0.62	0.64	0.56
B. Primary education	rank *	0.54	0.24	0.37	0.48	0.41	0.47	0.51	0.44
Health equality	scale (0 to 1)	0.51	0.32	0.45	0.58	0.41	0.54	0.58	0.52
Mortality rate, adult, male (per 1000 male adults)	value	−0.64	−0.32	−0.49	−0.60	−0.58	−0.57	−0.64	−0.59
B. Primary education	value	0.54	0.21	0.34	0.44	0.43	0.44	0.48	0.41
Country-code top-level domains (ccTLDs)	score (0–100)	0.41	0.31	0.48	0.58	0.42	0.57	0.65	0.60
Fertility rate, total (births per woman)	value	−0.55	−0.30	−0.47	−0.55	−0.50	−0.58	−0.64	−0.58
Access to electricity (% of population)	value	0.60	0.35	0.53	0.62	0.53	0.56	0.63	0.58
Knowledge and technology outputs	rank *	0.65	0.04	0.25	0.40	0.56	0.39	0.53	0.50
Access to electricity, urban (% of urban population)	value	0.65	0.29	0.47	0.55	0.52	0.51	0.58	0.52

* The correlations of the rank and some score variables were multiplied by −1.

**Table A3:** Standalone correlations in the *business and ICT* community. Spearman rank correlations between COVID-19 variables and indicators of the *business and ICT* community in the worldwide dataset.

Name	Type	dyssincefstcase	cases20dysaftfst	cases40dysaftfst	cases60dysaftfst	dyssincefstdeath	deaths20dysaftfst	deaths40dysaftfst	deaths60dysaftfst
Global Competitiveness Index	value	0.69	0.17	0.33	0.48	0.57	0.43	0.56	0.52
Laws relating to ICTs, 1–7 (best)	value	0.54	0.26	0.39	0.53	0.41	0.48	0.56	0.53
9th pillar: Economic impacts	value	0.61	0.23	0.41	0.55	0.54	0.52	0.63	0.59
Impact of ICTs on access to basic services, 1–7 (best)	value	0.56	0.26	0.39	0.52	0.41	0.48	0.55	0.50
7th pillar: Business usage	rank *	0.55	0.16	0.28	0.42	0.45	0.37	0.47	0.44
Availability of latest technologies, 1–7 (best)	value	0.52	0.27	0.40	0.53	0.44	0.53	0.60	0.56
GCI 4.0: Enabling environment component	rank *	0.66	0.26	0.43	0.58	0.51	0.54	0.64	0.58
10th pillar: Social impacts	value	0.61	0.21	0.36	0.49	0.46	0.44	0.53	0.49
GCI 4.0: Pillar 1: Institutions	score	0.56	0.26	0.40	0.51	0.43	0.47	0.54	0.48
Business-to-consumer Internet use, 1–7 (best)	rank *	0.65	0.14	0.31	0.45	0.51	0.41	0.54	0.51
Property rights score	value	0.53	0.30	0.43	0.56	0.42	0.54	0.61	0.56
ICTs and business model creation	rank *	0.63	0.11	0.31	0.46	0.54	0.40	0.52	0.49
GCI 4.0: Pillar 12: Innovation capability	rank *	0.72	0.12	0.33	0.49	0.61	0.43	0.58	0.55
ICTs and organizational model creation	rank *	0.65	0.08	0.26	0.41	0.51	0.37	0.50	0.47
2nd pillar: Business and innovation environment	value	0.54	0.32	0.44	0.57	0.43	0.54	0.61	0.54
Impact of ICTs on new organizational models, 1–7 (best)	value	0.59	0.14	0.29	0.44	0.45	0.39	0.50	0.48
ICT use for business-to-business transactions, 1–7 (best)	value	0.55	0.18	0.29	0.42	0.38	0.40	0.48	0.46
Internet access in schools, 1–7 (best)	value	0.54	0.33	0.43	0.53	0.40	0.52	0.57	0.50
Impact of ICTs on business models, 1–7 (best)	rank *	0.57	0.17	0.32	0.46	0.47	0.41	0.50	0.48
Local supplier quality, 1–7 (best)	rank *	0.55	0.20	0.38	0.53	0.59	0.51	0.62	0.56
Internet access in schools	1–7 best	0.55	0.28	0.39	0.49	0.45	0.46	0.51	0.45
GCI 4.0: 1.F Property rights	score	0.53	0.24	0.40	0.51	0.41	0.50	0.56	0.51
A. Transport infrastructure	value	0.66	0.08	0.25	0.42	0.56	0.39	0.52	0.50
Value chain breadth, 1–7 (best)	rank *	0.67	0.05	0.24	0.38	0.60	0.34	0.46	0.42
GCI 4.0: Interaction and diversity	rank *	0.60	0.09	0.26	0.40	0.47	0.34	0.45	0.40
GCI 4.0: Markets component	score	0.75	0.01	0.22	0.40	0.62	0.33	0.50	0.48
12th pillar Innovation	rank *	0.60	0.07	0.23	0.37	0.51	0.31	0.43	0.39
Quality of air transport infrastructure	rank *	0.53	0.22	0.36	0.48	0.49	0.47	0.55	0.51
Quality of overall infrastructure	1–7 best	0.55	0.24	0.37	0.49	0.47	0.47	0.55	0.50
Ease of doing business	dtf	0.60	0.17	0.32	0.45	0.46	0.43	0.52	0.46
Country credit rating, 0–100 (best) *	rank *	0.64	0.14	0.30	0.48	0.54	0.44	0.57	0.54
GCI 4.0: Pillar 11: Business dynamism	rank *	0.62	0.12	0.30	0.44	0.52	0.42	0.52	0.47
GCI 4.0: Efficiency of air transport services	rank *	0.57	0.18	0.35	0.47	0.49	0.43	0.51	0.48
8th pillar: Government usage	value	0.59	0.11	0.25	0.39	0.41	0.33	0.41	0.39
GCI 4.0: Pillar 9: Financial system	rank *	0.64	0.13	0.26	0.41	0.49	0.41	0.51	0.47
Control of international distribution, 1–7 (best)	rank *	0.64	0.07	0.25	0.40	0.54	0.38	0.49	0.46
Quality of scientific research institutions	rank *	0.58	0.10	0.27	0.40	0.54	0.37	0.50	0.46
A. Transport infrastructure	rank *	0.72	0.05	0.29	0.46	0.59	0.41	0.56	0.54
Capacity for innovation	rank *	0.55	0.04	0.19	0.32	0.50	0.25	0.37	0.33
Nature of competitive advantage, 1–7 (best)	rank *	0.54	0.15	0.26	0.37	0.43	0.34	0.41	0.36
GCI 4.0: Border clearance efficiency	score	0.56	0.13	0.28	0.42	0.45	0.40	0.52	0.49
State of cluster development	rank *	0.64	−0.06	0.12	0.30	0.49	0.23	0.37	0.35
GCI 4.0: Quality of land administration	rank *	0.51	0.28	0.44	0.53	0.43	0.50	0.56	0.52
Group Grievance	score	−0.54	−0.18	−0.32	−0.49	−0.51	−0.44	−0.56	−0.53
GCI 4.0: 7.A Domestic competition	rank *	0.55	0.10	0.25	0.39	0.42	0.31	0.40	0.35
Government Online Service Index, 0-1 (best)	rank *	0.64	0.06	0.24	0.40	0.49	0.36	0.48	0.47
Company spending on Research & Development	rank *	0.57	−0.02	0.12	0.26	0.48	0.21	0.34	0.31
GCI 4.0: Digital skills among population	rank *	0.59	0.18	0.32	0.41	0.45	0.37	0.42	0.36
GCI 4.0: Efficiency of seaport services	rank *	0.58	0.06	0.24	0.39	0.51	0.35	0.46	0.44
Getting electricity: Cost	% of income per capita	−0.62	−0.19	−0.32	−0.44	−0.57	−0.38	−0.49	−0.45
Degree of customer orientation, 1–7 (best)	value	0.54	0.11	0.26	0.39	0.54	0.33	0.43	0.38
Use of virtual social networks, 1–7 (best)	value	0.55	0.26	0.38	0.47	0.43	0.50	0.53	0.48
Registering property: Reliability of infrastructure index	0–8	0.53	0.23	0.39	0.49	0.41	0.47	0.54	0.51
Availability of scientists and engineers	rank *	0.64	0.00	0.17	0.29	0.57	0.26	0.39	0.34
A. Efficiency	value	0.54	0.11	0.17	0.32	0.36	0.27	0.36	0.35

* The correlations of the rank variables were multiplied by −1.

**Table A4:** Standalone correlations in the *regulation and corruption* community. Spearman rank correlations between COVID-19 variables and indicators of the *regulation and corruption* community in the worldwide dataset.

Name	Type	dyssincefstcase	cases20dysaftfst	cases40dysaftfst	cases60dysaftfst	dyssincefstdeath	deaths20dysaftfst	deaths40dysaftfst	deaths60dysaftfst
Regulatory quality	rank *	0.47	0.32	0.44	0.56	0.40	0.57	0.61	0.56
Political environment	score (0–100)	0.42	0.33	0.41	0.52	0.33	0.53	0.56	0.51
Institutions	score (0–100)	0.48	0.31	0.42	0.53	0.39	0.52	0.58	0.52
Freedom from corruption score	value	0.43	0.32	0.41	0.53	0.36	0.51	0.56	0.52
Government officials in the police and the military do not use public office for private gain	total	0.50	0.20	0.35	0.49	0.43	0.52	0.59	0.52
Due process of law and the rights of the accused	total	0.46	0.23	0.40	0.55	0.45	0.55	0.62	0.56
Government regulations are applied and enforced without improper influence	total	0.42	0.17	0.28	0.43	0.40	0.46	0.54	0.49
The right to life and security of the person is effectively guaranteed	total	0.32	0.30	0.47	0.61	0.38	0.60	0.65	0.59
Corruption	scale (0 to 1) *	−0.46	−0.21	−0.32	−0.47	−0.37	−0.48	−0.54	−0.49
Regulatory environment	score (0–100)	0.36	0.26	0.36	0.45	0.25	0.49	0.55	0.50
Trading across borders: Time to import	days	−0.55	−0.23	−0.38	−0.52	−0.45	−0.56	−0.62	−0.59
Civil Liberties	1 to 7 scale *	0.22	0.28	0.40	0.47	0.34	0.50	0.54	0.50
Freedom from arbitrary interference with privacy is effectively guaranteed	total	0.38	0.19	0.39	0.54	0.42	0.52	0.59	0.54
Uneven Development	score	−0.29	−0.28	−0.39	−0.47	−0.38	−0.48	−0.55	−0.52
Election government intimidation	scale (0 to 1) *	−0.34	−0.25	−0.36	−0.43	−0.42	−0.47	−0.54	−0.49
Election other voting irregularities	scale (0 to 1) *	−0.42	−0.23	−0.34	−0.45	−0.40	−0.47	−0.55	−0.51

* The correlations of the rank and scale variables were multiplied by −1.

**Table A5:** Standalone correlations in the *protectionism* community. Spearman rank correlations between COVID-19 variables and indicators of the *protectionism* community in the worldwide dataset.

Name	Type	dyssincefstcase	cases20dysaftfst	cases40dysaftfst	cases60dysaftfst	dyssincefstdeath	deaths20dysaftfst	deaths40dysaftfst	deaths60dysaftfst
Tariff rate, applied, simple mean, all products (%)	value	−0.28	−0.39	−0.48	−0.54	−0.29	−0.52	−0.55	−0.50
Tariff rate, most favored nation, weighted mean, manufactured products (%)	value	−0.40	−0.35	−0.45	−0.54	−0.35	−0.50	−0.56	−0.51
GCI 4.0: Trade tariffs	rank	−0.30	−0.36	−0.47	−0.56	−0.30	−0.52	−0.56	−0.51
Tariff rate, most favored nation, simple mean, manufactured products (%)	value	−0.36	−0.37	−0.47	−0.55	−0.32	−0.51	−0.56	−0.50
Tariff rate, most favored nation, weighted mean, all products (%)	value	−0.37	−0.32	−0.46	−0.56	−0.35	−0.55	−0.61	−0.56
Trade freedom score	value	0.39	0.34	0.44	0.51	0.34	0.53	0.55	0.49
Effectively applied import tariff rates on non−agricultural and non−fuel products	average	−0.34	−0.33	−0.42	−0.47	−0.36	−0.52	−0.56	−0.49
Share of tariff lines with international peaks, manufactured products (%)	value	−0.34	−0.39	−0.47	−0.56	−0.27	−0.50	−0.53	−0.49
